# The development of priority decision model for old urban community renovation in China

**DOI:** 10.1038/s41598-024-54883-3

**Published:** 2024-02-23

**Authors:** Wei Wang, Huawei Yang, Shuwei Jing

**Affiliations:** College of Management Science and Engineering, Shanxi University of Finance & Economics, No.140, Wucheng Road, Taiyuan, 030600 Shanxi China

**Keywords:** Civil engineering, Sustainability

## Abstract

Old urban community renovation is an important task of urban renewal in China. In order to ensure the quality and efficiency of renovation work, the government requires a method to assess the priority for old urban community renovation. This paper proposes an evaluation model from a more comprehensive perspective. It establishes the evaluation index system with CIPP model. The method of Cloud-VIKOR is selected to construct the evaluation model. Finally, selects nine case communities to verify the evaluation model. The results show that the evaluation index system covers the whole process of renovation project and the evaluation indexes proposed in the existing research. The priority decision result of 9 case communities is basically consistent with the actual renovation sequence and does not change greatly due to the fluctuation of decision-making mechanism coefficient or the evaluation index weight. This evaluation model can help decision-makers diagnose and optimize the renovation project.

## Introduction

Old urban community renovation is an important task of urban renewal in China. The Ministry of Housing and Urban–Rural Development selected 15 pilot cities for old urban community renovation in 2018. The purpose is to explore the patterns of old urban community renovation. The State Council issued *the Guiding Opinions on Comprehensively Promoting the Old Urban Community Renovation* in 2020. It is required to complete the renovation work of all the old urban communities built before 2000 in the 14th “Five-Year Plan” period. Subsequently, local governments issued corresponding policies and plans for old urban community renovation based on the regions' actual conditions. Old urban community renovation can not only improve the living conditions of residents, but also relate to the national urban development strategy.

There are three major problems in old urban community renovation, including huge stock, complex situation, and limited funds. First, the State Council Information Office announced that the number of old urban communities built before 2000 was about 219,000. Second, the objective conditions of old urban communities are different, such as the construction years, geographical environment, and maintenance status. There is obvious diversity and difference in the renovation needs of old urban communities. Third, the experts estimate that the capital demand of old urban community renovation will exceed 6 trillion yuan. Although the government has put forward many measures to raise the renovation funds, there are still great difficulties in raising funds for old urban community renovation.

In order to complete the work of old urban community renovation and ensure the quality and efficiency of renovation works, the government requires a method to arrange the renovation sequence for old urban communities. This method should evaluate the priority of old urban community renovation scientifically. Therefore, it is important to research the priority decision for old urban community renovation. This paper provides a method to evaluate the priority for old urban community renovation.

This research mainly includes the following three aspects: (1) Establish an evaluation index system using CIPP theory and Systematic Literature Review method; (2) Construct an evaluation model using the method of Cloud-VIKOR; (3) select 9 case communities to test that the evaluation model is scientific.

The overall structure of this paper is as follows. Section "Literature review" presents the literature review, which is a major retrospective of the evaluation methods for old urban community renovation. Section "The evaluation index system" describes the construction process of the evaluation index system. Section "The evaluation model" constructs the evaluation model using the method of Cloud-VIKOR. Section "Case study" selects nine case communities to verify that the evaluation method is scientific. Finally, conclusions and future research directions are presented in Section "Conclusion".

## Literature review

The existing research on evaluation methods for old urban community renovation can be classified into three types: urban planning evaluation, renovation scheme evaluation, and renovation performance evaluation.

The purpose of an urban planning evaluation is to identify the renovation priority for an urban area or an old urban community. In order to identify the renovation priority for urban areas, scholars select the Analytic Hierarchy Process (AHP) and the Geographic Information System (GIS) to assess the buildings in the city. For example, Foroughi & Rasol^1^ assess the buildings’ modernization level with AHP, and then use GIS to identify the renovation priority for urban areas,Haghighi Fard & Doratli^2^ assess the buildings’ resilience level with AHP, and then use GIS to identify the renovation priority for urban areas. Martí et al.^3^ assess the activity level for urban areas according to the user data of multiple Location Based Social Networks (LBSNs), and then determine the renovation priority of urban areas. In order to identify the renovation priority for old urban communities, some scholars assess the actual situation of old urban communities from different perspectives, such as the renovation potential^4^, the environmental efficiency^5^, and the renewal potential of land use^6^. Other scholars assess the renovation priority for old urban community based on the willingness of residents^7^. Andersen et al.^8^ use only the public accessible building data to identify the renovation priority for old urban communities and propose that the government should establish a national or regional digital building data register for old urban communities. In addition, Ruá et al.^9^ assess the renovation priority for old urban community from three aspects: the urban planning, the actual situation of the community and the status of the buildings.

The purpose of a renovation scheme evaluation is to design a renovation scheme for an old urban community. The renovation scheme evaluation mainly includes two aspects: the actual situation of old urban community and the demands of stakeholders. Knippschild & Zöllter^10^ analyze the Urban Transformation Matrix used in eastern Germany, which contains 12 renovation schemes. This Matrix assesses the actual status of buildings in four aspects and classifies the location of old urban community into three types. Some scholars analyze the residents’ satisfaction using different methods, such as the Structural Equation Modeling (SEM)^11^ and the Revised Importance-Performance Analysis (R-IPA)^12^, and then determine the renovation scheme for old urban community. Serrano-Jiménez et al.^13^ point out that there are always differences between the architectural demands and the residents’ perceptions when determining the renovation scheme, and propose the Architectural and Psycho-environmental Retrofitting Assessment Method (APRAM) for the renovation scheme evaluation. The demands of different stakeholders are also different when it comes to determining a renovation scheme. Bottero et al.^14^ use the methods of Novel Approach to Imprecise Assessment and Decision Environments (NAIADE) and Multi-Attribute Value Theory (MAVT) to determine a balanced renovation scheme. The results can meet the demands of most stakeholders. Bottero et al.^15^ also use the methods of SWOT analysis and PROMETHEE to integrate the demands of multiple stakeholders, and then evaluate the renovation schemes. In addition, Lee & Chan^16^ use AHP to evaluate the renovation scheme from the perspective of sustainability, which includes economic sustainability, environmental sustainability, and social sustainability.

The purpose of a renovation performance evaluation is to assess the results of an old urban community renovation. Zhang et al.^17^ use the Fuzzy Comprehensive Assessment Method to assess the renovation performance in two aspects: the buildings and the public facilities. Xiao et al.^18^ assess the renovation performance from more aspects, including buildings, public facilities, property management, and long-term management mechanism. Zhu et al.^19^ construct a comprehensively evaluation model with the method of AHP and TOPSIS, which evaluates the renovation performance in four aspects: the buildings performance, the economic performance, the environmental performance, and the social performance. In addition, Li et al.^20^ assess the renovation performance from the perspective of the input and output, and use the methods of Principal Component Analysis (PCA) and Data Envelopment Analysis (DEA) to construct the evaluation model. Lee & Lim^21^ analyze the difference in the evaluation indicators and weights for two types old urban community renovation projects, the economy-based and community-based renovation projects. The results show that the evaluation of renovation performance should be consistent with the renovation objectives and the communities’ background.

As mentioned above, in the research of urban planning evaluation, most scholars select the qualitative evaluation methods to assess the renovation priority, such as the AHP. The perspective of urban planning evaluation is mainly classified into two aspects, the situation of old urban community and the willingness of residents. In the research of renovation scheme evaluation, scholars select multiple evaluation methods to assess the renovation scheme for old urban community renovation, and the perspective mainly focuses on the actual conditions of the old urban community or the demands of stakeholders. In the research of renovation performance evaluation, scholars mainly select quantitative evaluation methods to assess the renovation performance. The evaluation index system mainly includes three aspects, namely social performance, economic performance, and environmental performance. However, the existing research is mostly focused on a single perspective, such as the community’s situation or residents’ willingness. Second, old urban community renovation project involves a series of phases from the start to the completion, but the existing research is only focus on a single phase, like urban planning, renovation scheme design, or renovation performance evaluation. Third, the existing research are mainly use the qualitative evaluation methods to assess the priority for old urban community renovation. Therefore, this paper hopes to construct an evaluation model from a comprehensive perspective and provides a scientific method to assess the priority for old urban community renovation.

## The evaluation index system

### CIPP theory and systematic literature review method

This paper uses the CIPP evaluation model to establish the evaluation index system, and the Systematic Literature Review method is used to determine the evaluation indexes in the evaluation index system.

#### CIPP theory

The CIPP evaluation model was firstly proposed by Stufflebeam in 1960s. Over the years, this model has widely applied to evaluate educational programs^22^. The core concepts of this model include four aspects: Context, Input, Process, and Product. It covers all the phases in a project from the start to the completion^23^. The model's underlying theme is that evaluation's most important purpose is not to prove, but to improve^24^, which means that the evaluation results should not only help decision makers to diagnose the project, but also provide support for decision makers to optimize the project^25^. The CIPP evaluation model can be applied in numerous fields^24^, such as education, healthcare, business, and construction. Wati et al.^26^ evaluate the Green Open Space Management Program in Gresik Regency with the CIPP evaluation model.

#### Systematic literature review method

The Systematic Literature Review method can help researchers sort out the relevant papers in a certain field comprehensively. This method mainly includes three steps: evaluation index collection, evaluation index analysis and evaluation index system construction^27^. The evaluation index collection includes three works: keywords determination, literature retrieval and screening, and evaluation indexes collection. The evaluation index analysis includes two works: sorting out the selected evaluation indexes and identifying the indexes adopted in the paper. The evaluation index system construction includes two works: classifying the indexes and constructing the evaluation index system.

### Evaluation index collection

According to the existing research on old urban community renovation, the keywords selected in this paper are as follows: old urban community, old urban community renovation, urban renewal, urban regeneration, urban renaissance, urban revitalization, urban redevelopment, and urban transformation. The databases selected for getting the relevant papers are China National Knowledge Internet (CNKI) and Web of Science. The process of literature retrieval and screening involves four steps:

*Step 1*: Search the papers since 2018;

*Step 2*: Exclude the irrelevant papers, book reviews, editorials, and newspapers;

*Step 3*: Exclude the papers without an evaluation index system;

*Step 4*: Supplement the papers published before 2018.

The literature retrieval and screening were completed in March 2022. This research finally obtained 23 papers, and these papers totally contain 388 evaluation indicators. After combining the same or similar evaluation indicators, 88 different evaluation indicators were initially extracted.

### Evaluation index analysis

Firstly, this paper summarizes the 88 evaluation indicators into 21 evaluation indexes. There are 20 evaluation indicators used to evaluate the conditions of buildings and communities, such as the buildings performance, spatial distribution, structure type, completeness of facilities, communities’ situation, community location, floor area ratio, land utilization ratio, green space ratio, landscape ratio, indoor and outdoor environment quality, building energy consumption, waste management, etc. Therefore, these indicators can be summarized as the Community's situation. There are 11 evaluation indicators used to evaluate the renovation contents involved in the renovation scheme, such as the architectural design, land-use planning, security design, elevator installation, barrier-free design, building energy conservation, infrastructure renovation, etc. Therefore, these indicators can be summarized as the Comprehensiveness of renovation scheme. There are 15 evaluation indicators used to evaluate the design concept of renovation scheme, such as the green design, multi-functionality, open spaces provision, mixed development, livability, sustainability, waste disposal, social housing provision supply, regional service capability, compatibility with neighborhood, etc. Therefore, these indicators can be summarized as the Progressiveness of renovation scheme. There are 6 evaluation indicators used to evaluate the renovation impact on the community value, such as the housing appreciation, employment increase, etc. Therefore, these indicators can be summarized as the Expected value of community. There are 11 evaluation indicators used to evaluate the renovation performance on buildings and facilities, such as the building renovation performance, infrastructure renovation performance, access to public facilities, public facilities provision etc. Therefore, these indicators can be summarized as the Expected renovation performance of buildings and facilities. There are 4 evaluation indicators used to evaluate the renovation performance on community environment, including air quality, climate, landscape quality, and green space quality. Therefore, these indicators can be summarized as the Expected renovation performance of landscapes and greening spaces. In addition, the Expected complexity of organizational management includes two indicators which are the number of participating units and manpower input; The Expected residents’ satisfaction includes two indicators which are residents’ satisfaction and sense of belongings on community; The capability of long-term management includes three indicators, namely standardized property management, autonomous organization cultivation, and daily management mechanism; The Guarantee of renovation funds includes three indicators, namely funding sources, participation mechanism establishment, mutually beneficial agreement. Above all, this paper finally determines 21 evaluation indexes for the priority decision on old urban community renovation.

### Evaluation index system construction

This paper classifies the 21 evaluation indexes with the CIPP evaluation model into four aspects: Context, Input, Process and Product. The Context evaluates the degree of compliance between the community’s actual situation and the policy standard for renovation. Among the 21 evaluation indexes, 6 evaluation indexes belong to the Context evaluation, including age of buildings, community’s population, community’s historical and cultural value, residents’ support for renovation, function as a role-model for other communities, and community’s situation. Three of them reflect the policy relevance, namely age of buildings, function as a role-model for other communities, and community’s historical and cultural value, while others reflect the urgency of renovation, namely community’s situation, community’s population, and residents’ support for renovation.

The Input evaluates the rationality of resources investment on old urban community renovation. Among the 21 evaluation indexes, 3 evaluation indexes belong to the Input evaluation, including expected renovation costs, expected renovation duration, and expected maintenance costs. Two of them belong to the expected renovation input, which are expected renovation costs and expected renovation duration, while the expected maintenance costs belong to expected maintenance input.

The Process evaluates the feasibility of implementation process for old urban community renovation. Among the 21 evaluation indexes, 6 evaluation indexes belong to the Process evaluation, including comprehensiveness of renovation scheme, progressiveness of renovation scheme, expected disturbance to residents’ lives, expected disturbance to buildings, expected complexity on organizational management, and guarantee of renovation funds. It is divided into three aspects, namely renovation scheme, renovation risk, and project management. The renovation scheme includes two indexes, namely the comprehensiveness of the renovation scheme and the progressiveness of renovation scheme; The renovation risk includes two indexes, namely expected disturbance to residents’ lives and expected disturbance to buildings; The project management includes two indexes, namely expected complexity on organizational management and guarantee of renovation funds.

The product evaluates the expected performance of old urban community renovation. Among the 21 evaluation indexes, 6 evaluation indexes belong to the Product evaluation, including capacity of long-term management, expected residents’ satisfaction, buildings’ expected service life, expected value of community, expected renovation performance of buildings and facilities, expected renovation performance of landscapes and greening spaces. It is also divided into three aspects, namely expected social performance, expected economic performance, and expected environmental performance. The expected social performance includes two indexes, namely capacity of long-term management and expected residents' satisfaction; The expected economic performance includes two indexes, namely buildings’ expected service life and expected value of community; The expected environmental performance includes two indexes, namely expected renovation performance of buildings and facilities, expected renovation performance of landscapes and greening spaces. The evaluation index system for old urban community renovation constructed in this paper is shown in Table 1.

**Table 1:** The evaluation index system for old urban community renovation.

First-level index	Secondary index	Third-level index	References
Context (*A*)	Policy relevance $({A}_{1})$	Age of buildings $({A}_{11})$	^1,2^
		Function as a role-model for other communities ($${A}_{12})$$	^10^
		Community’s historical and cultural value ($${A}_{13})$$	^41,16^
	The urgency of renovation $$({A}_{2})$$	Community’s situation ($${A}_{21})$$	^15,11,6^
		Community’s population $({A}_{22})$	^9,5^
		Residents' support for renovation ($${A}_{23})$$	^41,16^
Input (*B*)	Expected renovation input $({B}_{1})$	Expected renovation costs $({B}_{11})$	^13,20^
		Expected renovation duration $({B}_{12})$	^42^
	Expected maintenance input $({B}_{2})$	Expected maintenance costs $({B}_{21})$	^4^
Process (*C*)	Renovation scheme $({C}_{1})$	Comprehensiveness of renovation scheme $({C}_{11})$	^14,43^
		Progressiveness of renovation scheme $({C}_{12})$	^14,43^
	Renovation risk (${C}_{2}$)	Expected disturbance to residents’ lives $({C}_{21})$	^13,20^
		Expected disturbance to buildings $({C}_{22})$	^19^
	Project management($${C}_{3})$$	Expected complexity on organizational management $$({C}_{31}$$)	^44,20^
		Guarantee of renovation funds $({C}_{32})$	^21^
Product (*D*)	Expected social performance $({D}_{1})$	Capability of long-term management $({D}_{11})$	^20,43^
		Expected residents’ satisfaction $({D}_{12}$)	^12,43^
	Expected economic performance $({D}_{2})$	Buildings’ expected service life $({D}_{21})$	^4^
		Expected value of community $({D}_{22})$	^8,9^
	Expected environmental performance $({D}_{3})$	Expected renovation performance of buildings and facilities $({D}_{31})$	^8,43^
		Expected renovation performance of landscapes and greening spaces $({D}_{32}$)	^18,17^

## The evaluation model

### Theory

The Cloud model was firstly proposed by Li in 1995. It is mainly used to realize the bidirectional transformation between qualitative concept and quantitative data^28^. The Cloud model is generally described with three digital features and expressed as $C\left(Ex,En,He\right)$^29^. It reflects the fuzziness and randomness comprehensively for a qualitative concept^30^, the expectation $Ex$ represents the center value of the qualitative concept, the entropy $En$ represents the measure of fuzziness, and the hyper-entropy $He$ represents the measure of uncertainty^31^. The Cloud model is widely used in the methods of Multicriteria Decision Making (MCDM).

VIKOR is an important method of MCDM. This method focuses on ranking and selecting for a set of alternatives, and proposes compromise solution (one or more) for a problem with conflicting criteria^32^. The compromise solution is the alternatives which are the closest to the ideal, it guarantees the minimum of individual regret and keeps the maximization of group utility^33^.

The method of Cloud-VIKOR combines the Cloud model with VIKOR, and used to solve the MCDM problems with uncertain linguistic information. Gao et al.^34^ propose a method of Cloud-VIKOR for interval MCDM problems. It transforms the ordinary interval values into Clouds, and then identify the compromise solutions with a Cloud model distance measure formula. Li et al.^35^ propose a Cloud-VIKOR method which combines the Cloud model, the Possibility degree theory, and the VIKOR expansion method. It also transforms the Clouds into interval values, and then construct the Possibility degree matrix with VIKOR expansion method to make the decision. Mo et al.^36^ point out that the method of transforming the Cloud model into interval value is not consider the randomness for the qualitative concepts, and construct the Cloud distance matrix with the Cloud Hamming distance formula to identify the compromise solutions. The priority decision for old urban community renovation is a typical MCDM problem with uncertain linguistic information. Therefore, this paper constructs the evaluation model with the method of Cloud-VIKOR.

### Linguistic variables and corresponding clouds

The Golden Section Method is a common method to transform linguistic variables into Clouds. Xu & Wu^37^ analyze the limitation of traditional Golden Section Method, and then propose an improved method as shown in Table 2. Suppose the effective domain used to assess a decision problem is $\left[{X}_{min},{X}_{max}\right]$, and the numbers of linguistic variables is $n$.

**Table 2:** Improved golden section method^37^.

Cloud	Expectation $Ex$	Entropy $En$	Hyper-entropy $He$
${C}_{+\frac{\left(n-1\right)}{2}}\left({Ex}_{+\frac{\left(n-1\right)}{2}},{En}_{+\frac{\left(n-1\right)}{2}},{He}_{+\frac{\left(n-1\right)}{2}}\right)$	${X}_{max}$	$\frac{{En}_{+\frac{\left(n-3\right)}{2}}}{0.618}$	$\frac{{He}_{+\frac{\left(n-3\right)}{2}}}{0.618}$
	…		
${C}_{+2}\left({Ex}_{+2},{En}_{+2},{He}_{+2}\right)$	${Ex}_{+1}+0.382\left({X}_{max}-{Ex}_{+1}\right)$	$\frac{{En}_{+1}}{0.618}$	$\frac{{He}_{+1}}{0.618}$
${C}_{+1}\left({Ex}_{+1},{En}_{+1},{He}_{+1}\right)$	${Ex}_{0}+0.382\left({X}_{max}-{Ex}_{0}\right)$	$0.382\frac{\left({X}_{max}-{X}_{min}\right)}{6}$	$\frac{{He}_{0}}{0.618}$
${C}_{0}\left({Ex}_{0},{En}_{0},{He}_{0}\right)$	$\frac{{X}_{min}+{X}_{max}}{2}$	$0.618{En}_{+1}$	${He}_{0}$
${C}_{-1}\left({Ex}_{-1},{En}_{-1},{He}_{-1}\right)$	${Ex}_{0}-0.382\left({Ex}_{0}-{X}_{min}\right)$	$0.382\frac{\left({X}_{max}-{X}_{min}\right)}{6}$	$\frac{{He}_{0}}{0.618}$
${C}_{-2}\left({Ex}_{-2},{En}_{-2},{He}_{-2}\right)$	${Ex}_{-1}-0.382\left({Ex}_{-1}-{X}_{min}\right)$	$\frac{{En}_{-1}}{0.618}$	$\frac{{He}_{-1}}{0.618}$
${C}_{-\frac{\left(n-1\right)}{2}}\left({Ex}_{-\frac{\left(n-1\right)}{2}},{En}_{-\frac{\left(n-1\right)}{2}},{He}_{-\frac{\left(n-1\right)}{2}}\right)$	…	$\frac{{En}_{-\frac{\left(n-3\right)}{2}}}{0.618}$	$\frac{{He}_{-\frac{\left(n-3\right)}{2}}}{0.618}$
	$${X}_{min}$$		

### Integrated cloud and cloud distance

#### Integrated cloud calculation

The Integrated Cloud is composed with two or more Clouds in the same effective domain. For a decision problem, suppose the number of alternatives is $m$, the number of evaluation indexes is $n$. ${C}_{ij}\left({Ex}_{ij},{En}_{ij},{He}_{ij}\right)$ represents the Integrated Cloud of alternative *Ai* on the evaluation index ${B}_{j}$,$i\in \left(\mathrm{1,2},3,\bullet \bullet \bullet ,m\right),j\in \left(\mathrm{1,2},3,\bullet \bullet \bullet ,n\right)$. The Integrated Cloud formula^38^ is

1 $${C}_{ij}\left({Ex}_{ij},{En}_{ij},{He}_{ij}\right)=\left(\sum_{k=1}^{k}{s}_{k}{Ex}_{ij}^{k},\sqrt{\sum_{k=1}^{k}{\left({s}_{k}{En}_{ij}^{k}\right)}^{2}},\sqrt{\sum_{k=1}^{k}{\left({s}_{k}{He}_{ij}^{k}\right)}^{2}}\right) .$$

where $k$ is the number of the experts, ${s}_{k}$ is the weight of the experts.

#### Cloud distance calculation

Cloud distance is the distance between two Clouds in the same effective domain. Wang & Liu^39^ calculate the Cloud distance with the Cloud Hamming distance formula. Suppose $D\left({C}_{1},{C}_{2}\right)$ represents the Cloud distance between ${C}_{1}\left({Ex}_{1},{En}_{1},{He}_{1}\right)$ and ${C}_{2}\left({Ex}_{2},{En}_{2},{He}_{2}\right)$, the Cloud Hamming distance formula is:

2 $$D\left({C}_{1},{C}_{2}\right)=\left|\begin{array}{c}\left(1-\frac{{{En}_{1}}^{2}+{{He}_{1}}^{2}}{{{En}_{1}}^{2}+{{He}_{1}}^{2}+{{En}_{2}}^{2}+{{He}_{2}}^{2}}\right){Ex}_{1}\\ -\left(1-\frac{{{En}_{2}}^{2}+{{He}_{2}}^{2}}{{{En}_{1}}^{2}+{{He}_{1}}^{2}+{{En}_{2}}^{2}+{{He}_{2}}^{2}}\right){Ex}_{2}\end{array}\right|.$$

If $${En}_{1}={He}_{1}={En}_{2}={He}_{2}=0$$, $$D\left({C}_{1},{C}_{2}\right)=\left|{Ex}_{1}-{Ex}_{2}\right|$$.

### Cloud distance-entropy weight method

Entropy Weight method is able to measures the importance of evaluation indexes according to the difference between evaluation results among different alternatives^37^. The Cloud Distance-Entropy Weight method^31^ used in this paper is as follows.

*Step 1* Calculate the Integrated Clouds for all alternatives on each evaluate indexes with formula (1), and then obtain the Integrated Cloud matrix as,

$${\varvec{Y}}=\left(\begin{array}{cccc}{{\varvec{C}}}_{11}& {{\varvec{C}}}_{21}& \cdots & {{\varvec{C}}}_{1{\varvec{n}}}\\ {{\varvec{C}}}_{21}& {{\varvec{C}}}_{22}& \cdots & {{\varvec{C}}}_{2{\varvec{n}}}\\ \vdots & \vdots & \ddots & \vdots \\ {{\varvec{C}}}_{{\varvec{m}}1}& {{\varvec{C}}}_{{\varvec{m}}2}& \cdots & {{\varvec{C}}}_{{\varvec{m}}{\varvec{n}}}\end{array}\right)$$

*Step 2* Set the Optimal Cloud as ${C}^{*}\left({Ex}^{*},{En}^{*},{He}^{*}\right)$, calculate the Cloud distance between Integrated Clouds and Optimal Cloud using formula (2), then obtain the Cloud distance matrix as,

$${\varvec{D}} = \left( {\begin{array}{*{20}c} {{\varvec{D}}_{11} \left( {{\varvec{C}}_{11} ,{\varvec{C}}^{\user2{*}} } \right)} & {{\varvec{D}}_{12} \left( {{\varvec{C}}_{12} ,{\varvec{C}}^{\user2{*}} } \right)} & \cdots & {{\varvec{D}}_{{1{\varvec{n}}}} \left( {{\varvec{C}}_{{1{\varvec{n}}}} ,{\varvec{C}}^{\user2{*}} } \right)} \\ {{\varvec{D}}_{21} \left( {{\varvec{C}}_{21} ,{\varvec{C}}^{\user2{*}} } \right)} & {{\varvec{D}}_{22} \left( {{\varvec{C}}_{22} ,{\varvec{C}}^{\user2{*}} } \right)} & \cdots & {{\varvec{D}}_{{2{\varvec{n}}}} \left( {{\varvec{C}}_{{2{\varvec{n}}}} ,{\varvec{C}}^{\user2{*}} } \right)} \\ \vdots & \vdots & \ddots & \vdots \\ {{\varvec{D}}_{{{\varvec{m}}1}} \left( {{\varvec{C}}_{{{\varvec{m}}1}} ,{\varvec{C}}^{\user2{*}} } \right)} & {{\varvec{D}}_{{{\varvec{m}}2}} \left( {{\varvec{C}}_{{{\varvec{m}}2}} ,{\varvec{C}}^{\user2{*}} } \right)} & \cdots & {{\varvec{D}}_{{{\varvec{mn}}}} \left( {{\varvec{C}}_{{{\varvec{mn}}}} ,{\varvec{C}}^{\user2{*}} } \right)} \\ \end{array} } \right)$$

*Step 3* Construct the normalized matrix $M$.

$${\varvec{M}}=\left(\begin{array}{cccc}{{\varvec{M}}}_{11}& {{\varvec{M}}}_{12}& \cdots & {{\varvec{M}}}_{1{\varvec{n}}}\\ {{\varvec{M}}}_{21}& {{\varvec{M}}}_{22}& \cdots & {{\varvec{M}}}_{2{\varvec{n}}}\\ \vdots & \vdots & \ddots & \vdots \\ {{\varvec{M}}}_{{\varvec{m}}1}& {{\varvec{M}}}_{{\varvec{m}}2}& \cdots & {{\varvec{M}}}_{{\varvec{m}}{\varvec{n}}}\end{array}\right)$$

3 $${M}_{ij}=\frac{{D}_{ij}\left({C}_{ij},{C}^{*}\right)}{\sum_{i=1}^{m}{D}_{ij}\left({C}_{ij},{C}^{*}\right)},i\in \left(\mathrm{1,2},\cdots m\right),j\in \left(\mathrm{1,2},\cdots n\right)$$

*Step 4* Calculate the entropy values ${H}_{j}$,

4 $${H}_{j}=-\frac{1}{{\text{ln}}m}\sum_{j=1}^{n}{M}_{ij}{\text{ln}}{M}_{ij}$$

Step 5 Calculate the weight credit utility value ${d}_{j}$,

5 $${d}_{j}=1-{H}_{j} ,$$

*Step 6* Determine the weight of evaluation indexes.

6 $${\omega }_{j}=\frac{{d}_{j}}{\sum_{j=1}^{n}{d}_{j}},$$

### The cloud-VIKOR method

This paper calculates the Cloud distance with formula (2), and then identify the value of group utility, individual regret, and VIKOR index^36^.

*Step 1* Determine the positive ideal solutions and negative ideal solutions for benefit-type indexes and cost-type indexes.

The positive ideal solution: The benefit-type indexes, ${C}_{j}^{+}=max\left({C}_{ij}\right),j\in \left(\mathrm{1,2},\cdots ,n\right)$; the cost-type indexes, ${C}_{j}^{+}=min\left({C}_{ij}\right),j\in \left(\mathrm{1,2},\cdots ,n\right)$.

The negative ideal solution: The benefit-type indexes, ${C}_{j}^{-}=min\left({C}_{ij}\right),j\in \left(\mathrm{1,2},\cdots ,n\right)$; the cost-type indexes, ${C}_{j}^{-}=max\left({C}_{ij}\right),j\in \left(\mathrm{1,2},\cdots ,n\right)$.

The comparison rules for two Clouds ${C}_{1}\left({Ex}_{1},{En}_{1},{He}_{1}\right)$ and ${C}_{2}\left({Ex}_{2},{En}_{2},{He}_{2}\right)$ is,

If $${Ex}_{1}>{Ex}_{2}$$, then $${C}_{1}>{C}_{2}$$;

when ${Ex}_{1}={Ex}_{2}$, if ${En}_{1}<{En}_{2}$, then ${C}_{1}>{C}_{2}$;

when ${Ex}_{1}={Ex}_{2}$, and ${En}_{1}={En}_{2}$, if ${He}_{1}<{He}_{2}$, then ${C}_{1}>{C}_{2}$.

*Step 2* Calculate the value of group utility and individual regret with formula (2).

The group utility value ${H}_{i}$,$i\in \left(\mathrm{1,2},\cdots ,m\right)$

7 $${H}_{i}=\sum_{j=1}^{n}{\omega }_{j}\frac{D\left({C}_{ij},{C}_{j}^{+}\right)}{D\left({C}_{j}^{+},{C}_{j}^{-}\right)},i\in \left(\mathrm{1,2},\cdots m\right),j\in \left(\mathrm{1,2},\cdots n\right)$$

The individual regret value ${R}_{i}$, $i\in \left(\mathrm{1,2},\cdots ,m\right)$

8 $${R}_{i}=max\left\{{\omega }_{j}\frac{D\left({C}_{ij},{C}_{j}^{+}\right)}{D\left({C}_{j}^{+},{C}_{j}^{-}\right)}\right\},i\in \left(\mathrm{1,2},\cdots m\right),j\in \left(\mathrm{1,2},\cdots n\right)$$

*Step 3* calculate the VIKOR Index ${Q}_{i}$, $i\in \left(\mathrm{1,2},\cdots ,m\right)$,

9 $${Q}_{i}=\gamma \frac{{H}_{i}-{H}^{-}}{{H}^{+}-{H}^{-}}+\left(1-\gamma \right)\frac{{R}_{i}-{R}^{-}}{{R}^{+}-{R}^{-}}$$

where ${H}^{+}=max\left\{{H}_{i}\right\},{H}^{-}=min\left\{{H}_{i}\right\},{R}^{+}=max\left\{{R}_{i}\right\},{R}^{-}=min\left\{{R}_{i}\right\}$. $\gamma$ is the decision-making mechanism coefficient (weight of the group utility), and $1-\gamma$ is the weight of the individual regret, $\gamma \epsilon \left[\mathrm{0,1}\right]$. $\gamma =0.5$ is usually set to maximize the group utility and minimize the individual regret. When $\gamma >0.5$, it means that decision makers are more inclined to the group utility, when $\gamma <0.5$, the decision makers are more inclined to the individual regret.

### Determination of compromise solution

Rank all alternatives according to the value of ${Q}_{i}$, ${H}_{i}$, ${R}_{i}$ from smallest to largest. Suppose alternative ${A}_{1}$ is the first position in the alternatives ranked by ${Q}_{i}$, if ${A}_{1}$ satisfies the following two conditions, then it is the optimal solution.

Condition 1, $Q\left({A}_{2}\right)-Q\left({A}_{1}\right)\ge 1/\left(m-1\right)$, ${A}_{2}$ is the second position in the alternatives ranked by ${Q}_{i}$, *m* is the quantity of alternatives.

Condition 2, Alternative ${A}_{1}$ must also be at the first position when ranked by ${H}_{i}$ and ${R}_{i}$.

If only Condition 1 is not satisfied, the compromise solutions will composed by ${A}_{1},{A}_{2},\dots {A}_{t}$, where ${A}_{t}$ is obtained according to $Q\left({A}_{t}\right)-Q\left({A}_{1}\right)\ge 1/\left(m-1\right)$ for maximum $t$.

If only Condition 2 is not satisfied, alternative ${A}_{1}$ and ${A}_{2}$ are both the optimal solutions.

## Case study

According to the existing research, this paper constructs an evaluation model to assess the priority for old urban community renovation. In order to more clearly introduce the application of the evaluation model, this paper select nine old urban communities in L City, Shanxi Province as cases, and represent as ${I}_{1}$ ~ ${I}_{9}$.

### Case situation

The nine old urban communities were built in different years, with four of them being renovated in 2021 and the other five in 2022. This paper collects information about the nine old urban community renovation projects on the government website, including the construction years, renovation time, community’s population, gross floor area, renovation costs, renovation duration, renovation scheme, and renovation funds. Among the nine communities, the earliest was built in 1995 and the latest in 2009. The renovation schemes of nine case communities cover different aspects, such as buildings, infrastructures, public facilities, landscape and greening space, and smart facilities. The nine renovation projects involve seven types of renovation funds, including residents self-financing, community owner financing, contractor investment, local financial funds, state subsidy funds, state budgetary funds and state special funds. The main situation of nine case communities is shown in Table 8.

### The evaluation model for priority decision with Cloud-VIKOR

#### Data Collection and Processing

This paper invites eleven experts $({K}_{1}\sim {K}_{11})$ to evaluate the nine case communities. There are four experts who are government officials from urban planning department in L city. There are four experts who are managers from the construction companies that is responsible for the case communities’ renovation task. The remaining three experts are university professors who study with the related fields. Seven experts have senior professional titles, and the remaining four experts have intermediate professional titles. In terms of academic qualifications, four experts have doctor’s degree, and seven experts have master’s degree. The age of eleven experts is between 37 and 52. The experts evaluation results are shown in Table 9.

This research divides the linguistic variables into 5 levels, as very high (VH), high (H), medium (M), low (L) and very low (VL). The evaluation index system used in this research is shown in Table 1.

##### Clouds corresponding to linguistic variables

The clouds corresponding to linguistic variables is defined with the method in Table 2, and the results are show as Table 3. The effective domain is set to [0,10], and given ${H}_{0}$=0.10.

**Table 3:** The Clouds corresponding to linguistic variables.

Linguistic variables	Clouds	Digital features
VH	${C}_{+2}$	(10.00, 1.03, 0.26)
H	${C}_{+1}$	(6.91, 0.64, 0.16)
M	${C}_{0}$	(5.00, 0.39, 0.10)
L	${C}_{-1}$	(3.09, 0.64, 0.16)
VL	${C}_{-2}$	(0.00, 1.03, 0.26)

##### Integrated clouds calculation

Suppose the weight of eleven experts is equal, then obtain the Integrated Cloud matrix with formula (1) (Table 4).

**Table 4:** The integrated cloud matrix.

	${I}_{1}$	${I}_{2}$	${I}_{3}$	${I}_{4}$	${I}_{5}$
${A}_{11}$	(6.32,0.19,0.05)	(5.98,0.17,0.04)	(1.30,0.27,0.07)	(4.13,0.17,0.04)	(10.00,0.31,0.08)
${A}_{12}$	(7.86,0.24,0.06)	(7.97,0.24,0.06)	(1.69,0.25,0.06)	(4.31,0.16,0.04)	(8.07,0.25,0.06)
${A}_{13}$	(7.41,0.22,0.06)	(8.70,0.27,0.07)	(2.03,0.24,0.06)	(3.61,0.18,0.04)	(8.53,0.27,0.07)
${A}_{21}$	(4.83,0.17,0.04)	(1.58,0.26,0.07)	(7.47,0.22,0.06)	(4.48,0.14,0.04)	(0.28,0.30,0.08)
${A}_{22}$	(8.70,0.27,0.07)	(1.40,0.26,0.07)	(5.00,0.13,0.03)	(7.47,0.22,0.06)	(8.88,0.27,0.07)
${A}_{23}$	(2.14,0.24,0.06)	(1.02,0.28,0.07)	(1.40,0.26,0.07)	(8.60,0.26,0.07)	(5.35,0.13,0.03)
${B}_{11}$	(4.16,0.22,0.06)	(0.84,0.28,0.07)	(8.42,0.26,0.07)	(7.62,0.24,0.06)	(5.67,0.21,0.05)
${B}_{12}$	(5.45,0.25,0.06)	(1.97,0.24,0.06)	(5.52,0.14,0.04)	(6.04,0.16,0.04)	(6.36,0.31,0.08)
${B}_{21}$	(7.97,0.24,0.06)	(8.64,0.27,0.07)	(3.50,0.18,0.05)	(2.66,0.22,0.06)	(6.56,0.18,0.05)
${C}_{11}$	(4.02,0.18,0.05)	(0.56,0.29,0.07)	(5.17,0.16,0.04)	(8.03,0.24,0.06)	(1.97,0.24,0.06)
${C}_{12}$	(4.02,0.18,0.05)	(0.84,0.28,0.07)	(5.52,0.16,0.04)	(8.31,0.25,0.06)	(1.69,0.25,0.06)
${C}_{21}$	(6.60,0.19,0.05)	(9.16,0.28,0.07)	(1.12,0.27,0.07)	(5.17,0.14,0.04)	(6.39,0.18,0.04)
${C}_{22}$	(6.32,0.18,0.05)	(9.16,0.28,0.07)	(1.69,0.25,0.06)	(4.13,0.17,0.04)	(8.60,0.26,0.07)
${C}_{31}$	(5.87,0.17,0.04)	(8.70,0.27,0.07)	(0.84,0.28,0.07)	(4.48,0.16,0.04)	(9.72,0.30,0.08)
${C}_{32}$	(5.17,0.16,0.04)	(5.52,0.16,0.04)	(4.13,0.16,0.04)	(8.88,0.27,0.07)	(3.44,0.18,0.05)
${D}_{11}$	(5.52,0.16,0.04)	(9.44,0.29,0.07)	(2.53,0.22,0.06)	(7.75,0.23,0.6)	(5.69,0.15,0.04)
${D}_{12}$	(5.00,0.13,0.03)	(8.70,0.27,0.07)	(6.04,0.18,0.04)	(0.84,0.28,0.07)	(8.60,0.26,0.07)
${D}_{21}$	(5.00,0.15,0.04)	(8.42,0.26,0.07)	(4.83,0.14,0.04)	(1.69,0.25,0.06)	(8.03,0.24,0.06)
${D}_{22}$	(8.60,0.26,0.07)	(8.14,0.25,0.06)	(1.12,0.27,0.07)	(4.65,0.15,0.04)	(7.19,0.21,0.05)
${D}_{31}$	(1.47,0.27,0.07)	(1.30,0.27,0.07)	(5.52,0.16,0.04)	(9.44,0.29,0.07)	(3.44,0.18,0.05)
${D}_{32}$	(1.40,0.26,0.07)	(0.56,0.29,0.07)	(5.87,0.17,0.04)	(7.23,0.22,0.06)	(1.69,0.25,0.06)

	${I}_{6}$	${I}_{7}$	${I}_{8}$	${I}_{9}$	—
${A}_{11}$	(0.00,0.31,0.08)	(3.78,0.17,0.04)	(8.88,0.27,0.07)	(7.75,0.23,0.06)	
${A}_{12}$	(1.12,0.27,0.07)	(6.04,0.16,0.04)	(5.69,0.15,0.04)	(0.28,0.30,0.08)	
${A}_{13}$	(1.40,0.26,0.07)	(5.35,0.13,0.03)	(5.35,0.13,0.03)	(0.84,0.28,0.07)	
${A}_{21}$	(7.75,0.23,0.06)	(5.52,0.14,0.04)	(2.77,0.22,0.06)	(9.44,0.29,0.07)	
${A}_{22}$	(5.87,0.16,0.04)	(1.69,0.25,0.06)	(0.56,0.29,0.07)	(3.78,0.17,0.04)	
${A}_{23}$	(4.31,0.15,0.04)	(6.04,0.16,0.04)	(8.60,0.26,0.07)	(3.78,0.17,0.04)	
${B}_{11}$	(2.81,0.21,0.05)	(3.29,0.23,0.06)	(2.66,0.22,0.06)	(5.69,0.15,0.04)	
${B}_{12}$	(5.87,0.16,0.04)	(2.03,0.24,0.06)	(3.05,0.20,0.05)	(5.21,0.19,0.05)	
${B}_{21}$	(0.00,0.31,0.08)	(5.17,0.14,0.04)	(6.04,0.16,0.04)	(1.40,0.26,0.07)	
${C}_{11}$	(9.72,0.30,0.08)	(5.52,0.14,0.04)	(1.97,0.24,0.06)	(7.47,0.22,0.06)	—
${C}_{12}$	(10.00,0.31,0.08)	(4.65,0.13,0.03)	(2.31,0.23,0.06)	(7.75,0.23,0.06)	
${C}_{21}$	(2.25,0.23,0.06)	(2.77,0.22,0.06)	(8.31,0.25,0.06)	(1.69,0.25,0.06)	
${C}_{22}$	(1.12,0.27,0.07)	(5.00,0.13,0.03)	(6.32,0.18,0.05)	(1.12,0.27,0.07)	
${C}_{31}$	(1.69,0.25,0.06)	(4.65,0.13,0.03)	(6.56,0.18,0.05)	(1.40,0.26,0.07)	
${C}_{32}$	(0.84,0.28,0.07)	(8.60,0.26,0.07)	(7.30,0.21,0.05)	(0.56,0.29,0.07)	
${D}_{11}$	(0.28,0.30,0.08)	(7.75,0.23,0.06)	(1.12,0.27,0.07)	(4.13,0.16,0.04)	
${D}_{12}$	(1.40,0.26,0.07)	(8.60,0.26,0.07)	(3.78,0.18,0.05)	(1.12,0.27,0.07)	
${D}_{21}$	(1.69,0.25,0.06)	(8.60,0.26,0.07)	(2.14,0.24,0.06)	(1.12,0.27,0.07)	
${D}_{22}$	(3.26,0.19,0.05)	(4.65,0.13,0.03)	(5.35,0.15,0.04)	(0.84,0.28,0.07)	
${D}_{31}$	(8.31,0.25,0.06)	(5.35,0.13,0.03)	(3.78,0.18,0.05)	(7.47,0.22,0.06)	
${D}_{32}$	(9.72,0.30,0.08)	(5.35,0.13,0.03)	(4.13,0.18,0.05)	(7.47,0.22,0.06)	

#### Weight calculation

The evaluation indexes in this research are all belong to benefit-type indexes, and the optimal cloud is ${C}_{2}(\mathrm{10.00,1.03,0.26})$. The Cloud distance matrix is obtained with formula (2).

Then, this research normalizes the Cloud distance matrix with formula (3). The entropy values, weight credit utility values, and evaluation indexes weights are obtained with formula (4), (5), (6), the results are shown as Table 5.

**Table 5:** The entropy values, weight credit utility values and evaluation indexes weights.

	Entropy	Weight credit utility value	Evaluation indexes weight		Entropy	Weight credit utility value	Evaluation indexes weight
${A}_{11}$	0.9130	0.0870	0.0375	${C}_{21}$	0.8894	0.1106	0.0477
${A}_{12}$	0.8812	0.1188	0.0512	${C}_{22}$	0.8732	0.1268	0.0547
${A}_{13}$	0.8745	0.1255	0.0541	${C}_{31}$	0.8688	0.1312	0.0566
${A}_{21}$	0.9084	0.0916	0.0395	${C}_{32}$	0.8808	0.1192	0.0514
${A}_{22}$	0.8738	0.1262	0.0544	${D}_{11}$	0.8925	0.1075	0.0463
${A}_{23}$	0.8854	0.1146	0.0494	${D}_{12}$	0.8494	0.1506	0.0649
${B}_{11}$	0.9085	0.0915	0.0394	${D}_{21}$	0.8674	0.1326	0.0572
${B}_{12}$	0.9547	0.0453	0.0195	${D}_{22}$	0.8876	0.1124	0.0484
${B}_{21}$	0.9096	0.0904	0.0390	${D}_{31}$	0.9096	0.0904	0.0390
${C}_{11}$	0.8928	0.1072	0.0462	${D}_{32}$	0.8764	0.1236	0.0533
${C}_{12}$	0.8835	0.1165	0.0502			Total	1.0000

#### VIKOR index calculation

According to the Integrated Cloud matrix in Table 4, the positive ideal solutions and negative ideal solutions are identified with the method in Table 3. The decision-making mechanism coefficient is set to 0.5. Calculate the group utility values and individual regret values with formula (7) and (8), then obtain the VIKOR indexes with formula (9). The results are shown as Table 6.

**Table 6:** The group utility value, individual regret value and VIKOR index.

	Group Utility Value	Ranking	Individual Regret Value	Ranking	VIKOR Index	Ranking
${I}_{1}$	0.3668	3	0.0410	3	0.1771	2
${I}_{2}$	0.4207	5	0.0533	4	0.4828	4
${I}_{3}$	0.6155	7	0.0572	7	0.8200	8
${I}_{4}$	0.3569	2	0.0649	9	0.6186	6
${I}_{5}$	0.2690	1	0.0395	2	0.0152	1
${I}_{6}$	0.6239	8	0.0562	6	0.8127	7
${I}_{7}$	0.4423	6	0.0387	1	0.2338	3
${I}_{8}$	0.4081	4	0.0544	5	0.4869	5
${I}_{9}$	0.6395	9	0.0602	8	0.9094	9

#### Renovation priority determination

According to the results shown in Table 6, community ${I}_{5}$ is the first position in the nine communities ranked by VIKOR indexes, and community ${I}_{1}$ is the second position.

Condition 1: ${I}_{1}-{I}_{5}=0.1771-0.0152=0.1619>\frac{1}{9-1}=0.125$. Condition 1 is satisfied.

Condition 2: community ${I}_{5}$ is the second position in the nine communities ranked by individual regret value. Condition 2 is not satisfied.

Therefore, community ${I}_{5}$ and ${I}_{1}$ are the optimal solutions, and the renovation sequence of nine old urban communities are ${I}_{5}$, ${I}_{1}$, ${I}_{7}$, ${I}_{2}$, ${I}_{8}$, ${I}_{4}$, ${I}_{6}$, ${I}_{3}$, ${I}_{9}$.

### Sensitivity analysis

In order to prove the evaluation model using Cloud-VIKOR which is proposed in this paper to be scientific, the antijamming ability of this model needs to be checked. In this research, the sensitivity analysis is approached from two aspects^40^: the decision-making mechanism coefficient $\gamma$, and the evaluation index weight ${\omega }_{j}$.

#### Sensitivity analysis on decision-making mechanism coefficient

**Figure 1:**
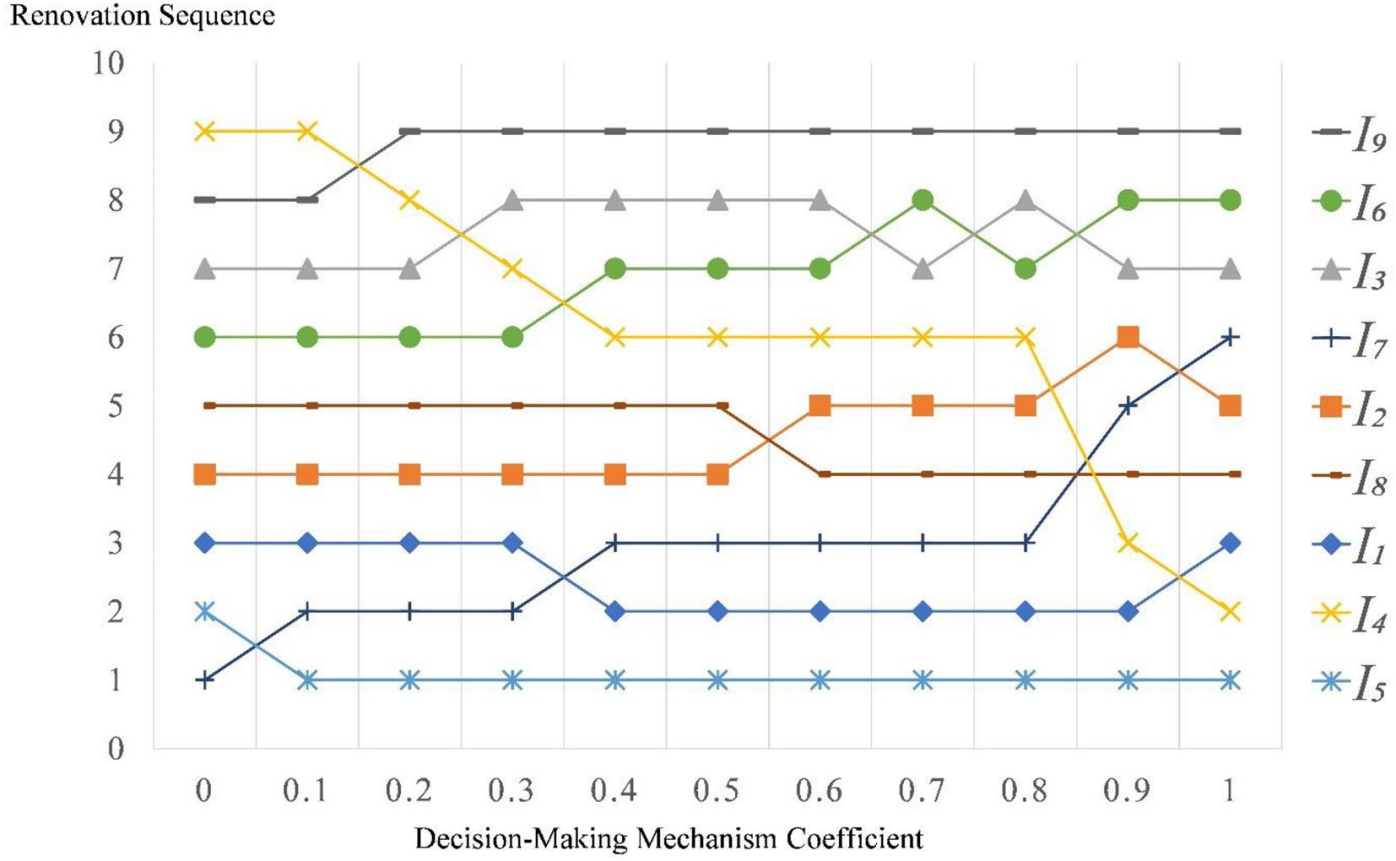
Sensitivity analysis on decision-making mechanism coefficient.

Let the decision-making mechanism coefficient change in the interval [0, 1] with a range of 0.1 each time, the results are shown in Fig. 1.

Figure 1 shows that, with the decision-making mechanism coefficient changing from 0.1 to 1.0, community ${I}_{5}$ takes the first position in the nine case communities, and with the decision-making mechanism coefficient changes from 0.1 to 0.8, the renovation sequence is not change greatly due to the fluctuation of decision-making mechanism coefficient.

#### Sensitivity analysis on evaluation index weight

**Figure 2:**
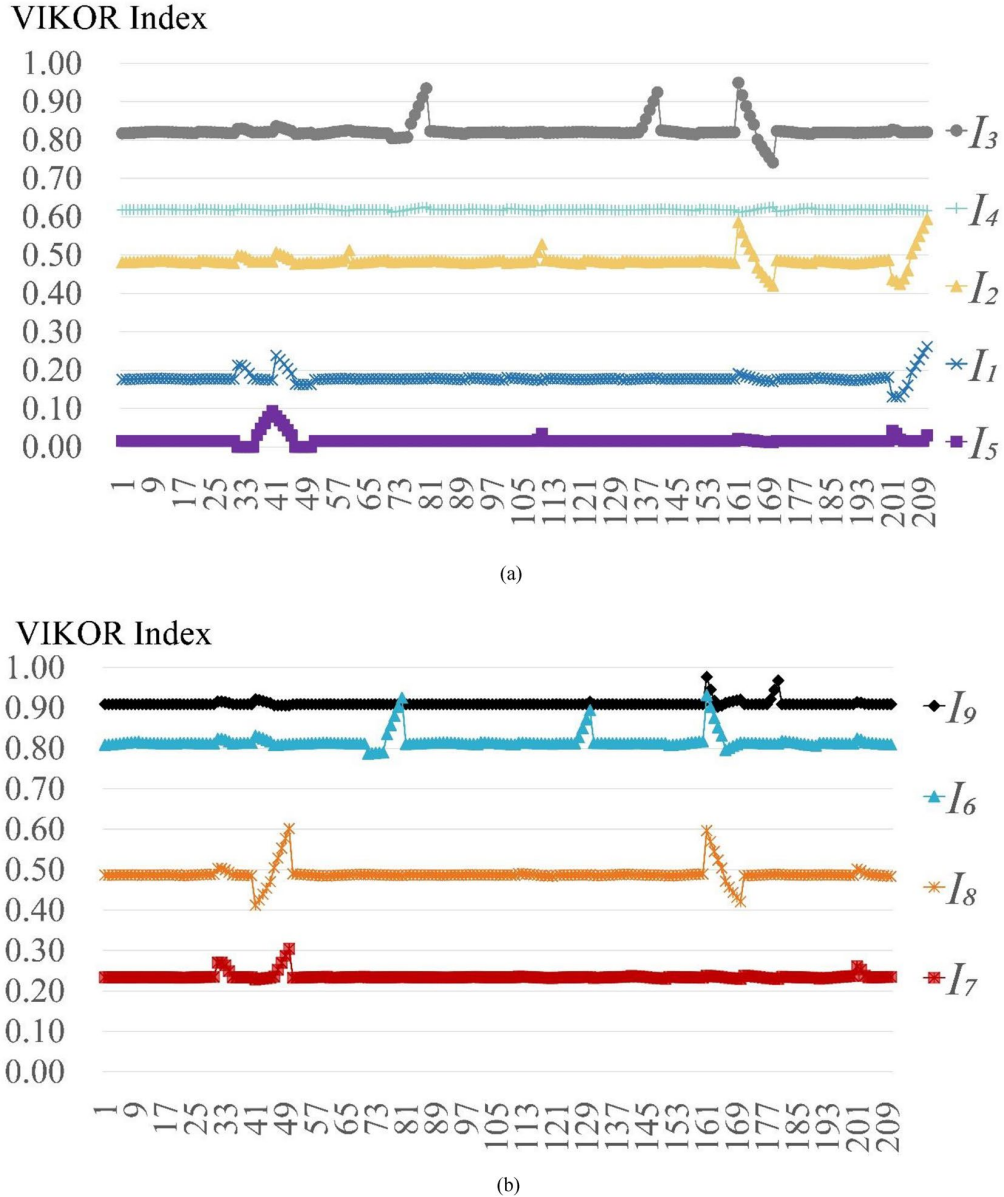
Sensitivity analysis on evaluation index weight.

This paper analyzes the sensitivity of evaluation index weights with Perturbation Method. Let the evaluation index weights change in the interval [− 10%, + 10%] with a range of 2% each time. Let the perturbation parameter be $\delta$, and $\delta$ will change within the interval [0.9, 1.1] with a range of 0.02 each time. When an evaluation index weight changes from ${\omega }_{j}$ to ${\omega }_{j}^{,}$, then ${\omega }_{j}^{,}=\delta {\omega }_{j}$, the rest of evaluation index weights will change with the coefficient $\theta$, and $\theta =(1- \delta {\omega }_{j})/(1-{\omega }_{j})$. This paper totally conducts 210 experiments for the 21 evaluation indexes, and the results is shown as Fig. 2.

Figure 2 shows that the renovation sequence of nine case communities is not change greatly due to the fluctuation of evaluation index weights. The community ${I}_{5}$ is always the optimal solution in the nine case communities within the 210 experiments. The renovation sequence of nine case communities remains unchanged for 184 times within the 210 experiments.

### Results

Firstly, the priority decision result of nine case communities is basically consistent with the actual renovation sequence. The priority decision result in Table 6 shows that the renovation sequence of nine case communities is ${I}_{5},{I}_{1},{I}_{7},{I}_{2},{I}_{8},{I}_{4},{I}_{6},{I}_{3},{I}_{9}$. The actual renovation sequence is ${I}_{5},{I}_{1},{I}_{2},{I}_{8},{I}_{7},{I}_{4},{I}_{6},{I}_{9},{I}_{3}$. The evaluation model using Cloud-VIKOR which is proposed in this paper is able to obtain an effective priority decision result for old urban community renovation.

Secondly, according to the results of sensitivity analysis, the renovation sequence of nine case communities does not change greatly due to the fluctuation of decision-making mechanism coefficient and evaluation index weight. It indicates that the priority decision results will not be affected by the decision makers' preference and the fluctuation of evaluation index weight. Therefore, the evaluation model proposed in this paper is reliable.

Thirdly, according to the theory of CIPP evaluation model, the evaluation model proposed in this paper should be able to help decision makers to diagnose and optimize the projects of old urban community renovation. Therefore, this paper analyzes the evaluation results in Table 4 from four aspects, that is Context, Input, Process, and Product. The results are shown in Table 7.

**Table 7:** The evaluation result analysis.

CIPP theory	Evaluation purpose	Analysis results		
		Best	Better	General
Context	The degree of conformity with the policy standards	${I}_{1}$, ${I}_{5}$,${I}_{7}$	${I}_{2}$, ${I}_{4}$, ${I}_{6}$,${I}_{8}$	${I}_{3}$, ${I}_{9}$
Input	The rationality of renovation input	${I}_{1}$, ${I}_{5}$	${I}_{2}$, ${I}_{3}$, ${I}_{4}$, ${I}_{7}$, ${I}_{8}$,${I}_{9}$	${I}_{6}$
Process	Feasibility of the renovation process	${I}_{4}$, ${I}_{7}$	${I}_{1}$, ${I}_{2}$, ${I}_{5}$,${I}_{8}$	${I}_{3}$, ${I}_{6}$,${I}_{9}$
Product	Expected renovation performance	${I}_{7}$	${I}_{1}$, ${I}_{2}$, ${I}_{3}$,${I}_{5}$	${I}_{4}$, ${I}_{6}$, ${I}_{8}$,${I}_{9}$

In the aspect of Context, Cases ${I}_{1}$, ${I}_{5}$ are given a higher renovation priority in most indexes, while Cases ${I}_{3}$, ${I}_{9}$ are given a lower renovation priority in most indexes. This result indicates that the conditions of Cases ${I}_{1}$, ${I}_{5}$ are highly in line with the policy standards of old urban community renovation, while the conditions of Cases ${I}_{3}$, ${I}_{9}$ are partially in line with the policy standards of renovation. Case ${I}_{7}$ is given a medium renovation priority in both aspects, policy relevance and renovation urgency. It indicates that the conditions of Case ${I}_{7}$ is also highly in line with the policy standards of renovation. Cases ${I}_{2}$, ${I}_{8}$ are given a higher renovation priority in terms of policy relevance, but a lower renovation priority in terms of renovation urgency, while Cases ${I}_{4}$, ${I}_{6}$ are opposite to Cases ${I}_{2}$, ${I}_{8}$. This result indicates that the conditions of Cases ${I}_{2}$, ${I}_{4}$, ${I}_{6}$, ${I}_{8}$ are basically in line with the policy standards of renovation. Therefore, in the aspect of Context, Cases ${I}_{1}$, ${I}_{5}$, ${I}_{7}$ are highly in line with the policy standards of old urban community renovation, while Cases ${I}_{2}$, ${I}_{4}$, ${I}_{6}$, ${I}_{8}$ are basically in line with the policy standards, and Cases ${I}_{3}$, ${I}_{9}$ are partially in line with the policy standards. Obviously, the evaluation results obtained in this paper are basically consistent with the actual situation of nine case communities.

In the aspect of Input, Cases ${I}_{1}$, ${I}_{5}$ are given a higher renovation priority compared with other case communities, while Case ${I}_{6}$ are given a lower renovation priority in most indexes. This result indicates that Cases ${I}_{1}$, ${I}_{5}$ have a best rationality in terms of renovation input, while Case ${I}_{6}$ has a general rationality. Cases ${I}_{3}$, ${I}_{4}$, ${I}_{9}$ are given a higher renovation priority in terms of expected renovation costs and a lower renovation priority in terms of expected maintenance costs. Cases ${I}_{2}$, ${I}_{7}$, ${I}_{8}$ are opposite to Cases ${I}_{3}$, ${I}_{4}$, ${I}_{9}$. This result indicates that Cases ${I}_{3}$, ${I}_{4}$, ${I}_{9}$ have a best rationality only in terms of expected maintenance costs, while Cases ${I}_{2}$, ${I}_{7}$, ${I}_{8}$ have a best rationality only in terms of expected renovation costs. Therefore, Cases ${I}_{2}$, ${I}_{3}$, ${I}_{4}$, ${I}_{7}$, ${I}_{8}$, ${I}_{9}$ are given a better rationality in terms of renovation input. To sum up, in the aspect of Input, the rationality of renovation input is best in Cases ${I}_{1}$, ${I}_{5}$, better in Cases ${I}_{2}$, ${I}_{3}$, ${I}_{4}$, ${I}_{7}$, ${I}_{8}$, ${I}_{9}$, and general in Case ${I}_{6}$.

In the aspect of Process, Cases ${I}_{4}$, ${I}_{7}$ are given a higher renovation priority in most indexes. This result indicates that Cases ${I}_{4}$, ${I}_{7}$ have a best feasibility in terms of renovation process. Case ${I}_{1}$ is given a medium renovation priority in most indexes. This result indicates that Case ${I}_{1}$ has a better feasibility in terms of renovation process. Cases ${I}_{2}$, ${I}_{5}$, ${I}_{8}$ are given a lower renovation priority in terms of renovation scheme, but were given a higher renovation priority in terms of renovation risk and project management. It is due to the renovation schemes of Cases ${I}_{2}$, ${I}_{5}$, ${I}_{8}$ are simple, and lack sufficient comprehensiveness and progressiveness. These renovation schemes have small impact on residents' lives and buildings, the renovation risks and difficulty on project management are also low. In addition, the renovation funds of Case ${I}_{5}$ lack sufficient guarantee. Therefore, the feasibility of renovation process in Cases ${I}_{2}$, ${I}_{5}$, ${I}_{8}$ is better. Cases ${I}_{3}$, ${I}_{6}$, ${I}_{9}$ are given a higher renovation priority only in terms of renovation scheme, and a lower renovation priority in terms of renovation risk and project management. This result indicates that the renovation schemes of Cases ${I}_{3}$, ${I}_{6}$, ${I}_{9}$ are comprehensiveness and progressiveness. But on the one hand, these renovation schemes have a great impact on residents' lives and buildings, there are great risks in the renovation process. On the other hand, these renovation schemes need several construction parties in different professions, and the management work is more complicated. The renovation funds of these cases also lack sufficient guarantee. Therefore, the feasibility of renovation process in Cases ${I}_{3}$, ${I}_{6}$, ${I}_{9}$ is general. To sum up, in the aspect of process, the feasibility of renovation process is best in Cases ${I}_{4}$, ${I}_{7}$,better in Cases ${I}_{1}$, ${I}_{2}$, ${I}_{5}$, ${I}_{8}$, and general in Cases ${I}_{3}$, ${I}_{6}$, ${I}_{9}$.

In the aspect of Product, Case ${I}_{7}$ is given a higher renovation priority in most indexes. It is due to Case ${I}_{7}$ is given a higher renovation priority in both the Context and Process, so Case ${I}_{7}$ can obtain a best performance in expected renovation performance. Case ${I}_{8}$ is given a lower renovation priority in most indexes. It is because although the conditions of Case ${I}_{8}$ is basically in line with the policy standards of renovation, it is given a lower renovation priority in terms of the Input and Process, so the expected renovation performance of Case ${I}_{8}$ is general. Case ${I}_{3}$ is given a medium renovation priority in most indexes, because Case ${I}_{3}$ has high feasibility in terms of the Input, so it can obtain a better performance in expected renovation performance. Cases ${I}_{1}$, ${I}_{2}$, ${I}_{5}$ are given a higher priority in terms of social and economic performance, but are given a lower priority in terms of environmental performance. It is because Cases ${I}_{1}$, ${I}_{2}$, ${I}_{5}$ are highly in line with the policy standards of renovation, so these cases can obtain a good expected social and economic performance. But the renovation scheme lacks comprehensiveness and progressiveness, it cannot effectively improve the living environment. Therefore, the expected renovation performance of Cases ${I}_{1}$, ${I}_{2}$, ${I}_{5}$ is better. The evaluation results of Cases ${I}_{4}$, ${I}_{6}$, ${I}_{9}$ are opposite to those of Cases ${I}_{1}$, ${I}_{2}$, ${I}_{5}$. It is because Cases ${I}_{4}$, ${I}_{6}$, ${I}_{9}$ are not reasonable in terms of renovation input, so these cases are given lower renovation priority in terms of social and economic performance. The renovation schemes of Cases ${I}_{4}$, ${I}_{6}$, ${I}_{9}$ have good comprehensiveness and progressiveness, so the environmental performance is given a high renovation priority. Therefore, the expected renovation performance of Cases ${I}_{4}$, ${I}_{6}$, ${I}_{9}$ is general. To sum up, in the aspect of Product, the expected renovation performance is best in Case ${I}_{7}$, better in Cases ${I}_{1}$, ${I}_{2}$, ${I}_{3}$, ${I}_{5}$, and general in Cases ${I}_{4}$, ${I}_{6}$, ${I}_{8}$, ${I}_{9}$.

According to the analysis results, the top three Cases ${I}_{5}$, ${I}_{1}$, ${I}_{7}$ are highly in line with the policy standards of old urban community renovation, and have better performance in input rationality, renovation process feasibility, and expected renovation performance. Therefore, Cases ${I}_{5}$, ${I}_{1}$, ${I}_{7}$ are given a high priority for renovation. Cases ${I}_{5}$, ${I}_{1}$ are the most in line with the policy standards of renovation and have the most reasonable input, while Case ${I}_{7}$ is most feasible in the renovation process and can obtain the best renovation performance. Cases ${I}_{2}$, ${I}_{8}$, ${I}_{4}$, ${I}_{6}$, which rank from fourth to seventh, are all basically in line with the policy standards of renovation. Case ${I}_{2}$ has better performance in input rationality, renovation process feasibility, and expected renovation performance, while Case ${I}_{6}$ has general performance in input rationality, renovation process feasibility and expected renovation performance. Therefore, Cases ${I}_{2}$, ${I}_{8}$, ${I}_{4}$, ${I}_{6}$ are given a medium priority for renovation. The last two cases are ${I}_{3}$, ${I}_{9}$. These cases are basically in line with the policy standards. Although these cases have better performance in input rationality, the renovation process feasibility performance is general. In terms of expected renovation performance, Case ${I}_{3}$ is better and Case ${I}_{9}$ is general. Therefore, Cases ${I}_{3}$, ${I}_{9}$ are given a low priority for renovation. Above all, the priority decision results obtained with the evaluation model construct in this paper is reasonable.

Finally, this paper diagnoses the cases given low priority for renovation, and puts forward optimization suggestions. The renovation schemes of these cases include many contents, like building repair, building facilities renovation, infrastructure renovation, public facilities renovation, service facilities renovation, and greening renovation, which need several construction parties in different professions. In addition, these cases have smaller populations and less support from residents for the renovation. Therefore, the expected maintenance cost is higher, and the renovation risks and management difficulty are also greater. It is difficult to obtain good social and economic performance. In response to the above problems, this paper puts forward the optimization suggestions as follows:

1. Reduce the expected maintenance costs. The renovation schemes of these cases need to be optimized in terms of expected maintenance costs. Designers may choose materials and equipment with good durability and easy maintenance in the renovation schemes to reduce the costs of daily maintenance, and introduce intelligent technology, such as smart home systems, intelligent property management systems, etc., to improve the efficiency of maintenance management.
2. Improve the project management. Decision makers may consider implementing the renovation schemes in stages to reduce the difficulty on organization and management during the renovation process, and setting up a special project management team to deal with the challenges in communication and cooperation for multiple professional construction units.
3. Increase the residents' support for the renovation. Decision-makers can organize various publicity activities to raise residents' awareness on the necessity and benefits of renovation, and provide detail information in renovation plan to answer residents' doubts and concerns, to obtain their support and cooperation. In addition, feedback mechanisms can be set up to collect and process the residents' opinions and suggestions in a timely manner.

As mentioned above, the evaluation model using Cloud-VIKOR which is proposed in this paper is not only able to obtain a reasonable and effective priority decision result for old urban community renovation, but also can provide support for decision makers to diagnose and optimize the renovation project.

## Conclusion

The government requires a scientific method to make a priority decision for old urban community renovation, which is important to ensure the quality and efficiency for the renovation projects. This research proposes a scientific method to make the priority decision for old urban community renovation.

Firstly, this paper establishes an evaluation index system from the perspective of project whole process management, which covers the whole process of old urban community renovation project and the indexes proposed in the existing research. This research refines the problem that the existing researches only focus on a single perspective when evaluating the project of old urban community renovation, and provides reference for other countries and regional governments to formulate decision-making standards on old urban community renovation.

Secondly, this paper constructs the evaluation model using the method of Cloud-VIKOR and verified it with nine case communities. The priority decision result of nine case communities show that the evaluation model is not only able to obtain a scientific priority decision result for old urban community renovation, but also can help decision makers to diagnose and optimize the renovation projects. This provides a new decision-making method on the priority decision for old urban community renovation, and offers help for other countries and regional governments to formulate renovation plans on old urban community renovation.

There are still some limitations in this research. On the one hand, this paper only selects 9 old urban communities in L city to test that the evaluation model is scientific. In future research, it still needs to select more old urban communities in different cities to verify that this evaluation model is scientific. On the other hand, this research assumes that the evaluation weights of eleven experts from different units are the same. In the follow-up research, this research will analyze the importance of decision makers in the priority decision-making for old urban community renovation.

## Data Availability

All data generated or analysed during this research are included in this published paper.

## Appendix

See Appendix Tables 8 and 9.

**Table 8:** The situation of nine case communities.

Community	Renovation data	Built year	Community’s population (households)	Total building area (${m}^{2}$)	Renovation costs (Million yuan)	Renovation duration (days)	Source of renovation funds	The main content of renovation scheme	
${I}_{1}$	2021.3.2	2000	1216	127,729.13	2997.48	270	State special funds Local financial funds Community owner financing	Building repair Infrastructure renovation Service facilities renovation	Building facilities renovation Public facilities renovation
${I}_{2}$	2021.6.9	2001	211	21,414.00	526.93	180	State special funds Local financial funds Community owner financing	Building repair Infrastructure renovation	Public facilities renovation
${I}_{3}$	2022.5.30	2006	400	49,935.52	4228.91	365	State budgetary funds Contractor investment Residents self-financing	Building repair Infrastructure renovation Service facilities renovation	Building facilities renovation Public facilities renovation Greening renovation
${I}_{4}$	2022.2.24	2002	731	78,064.16	4598.07	365	Residents self-financing Contractor investment State subsidy funds	Building repair Infrastructure renovation Service facilities renovation Smart community renovation	Building facilities renovation Public facilities renovation Greening renovation
${I}_{5}$	2021.1.18	1995	836	95,522.00	2787.10	120	State budgetary funds Contractor investment Residents self-financing	Infrastructure renovation Greening renovation	Public facilities renovation
${I}_{6}$	2022.5.7	2005	444	57,293.68	2609.99	365	State budgetary funds Contractor investment Residents self-financing	Building repair Infrastructure renovation Service facilities renovation	Building facilities renovation Public facilities renovation Greening renovation
${I}_{7}$	2022.2.22	2000	216	23,790.68	1421.76	270	Residents self-financing Contractor investment State subsidy funds	Building repair Infrastructure renovation Service facilities renovation	Building facilities renovation Public facilities renovation Greening renovation
${I}_{8}$	2021.10.25	1998	173	11,688.45	591.23	90	Residents self-financing Contractor investment State subsidy funds	Building repair Infrastructure renovation	Public facilities renovation Greening renovation
${I}_{9}$	2022.5.13	1998	348	46,046.07	2713.43	365	State budgetary funds Contractor investment Residents self-financing	Building repair Infrastructure renovation Service facilities renovation	Building facilities renovation Public facilities renovation Greening renovation

**Table 9:** Experts rating results (linguistic variables).

Experts:${K}_{1}$		Institution: Government			Title: Senior titles			Degree: Master	
	${I}_{1}$	${I}_{2}$	${I}_{3}$	${I}_{4}$	${I}_{5}$	${I}_{6}$	${I}_{7}$	${I}_{8}$	${I}_{9}$
${A}_{11}$	M	M	VL	L	VH	VL	L	H	H
${A}_{12}$	VH	H	VL	L	M	L	H	M	VL
${A}_{13}$	H	H	VL	L	VH	L	M	M	VL
${A}_{21}$	L	VL	H	M	VL	H	M	L	VH
${A}_{22}$	VH	VL	M	H	H	M	L	VL	L
${A}_{23}$	L	VL	VL	H	M	M	H	VH	L
${B}_{11}$	VL	VL	VH	M	L	L	H	M	H
${B}_{12}$	L	L	H	M	VL	H	M	VL	VH
${B}_{21}$	M	VH	L	VL	H	VL	M	H	L
${C}_{11}$	L	VL	M	H	VL	VH	M	L	H
${C}_{12}$	L	VL	M	H	L	VH	M	VL	H
${C}_{21}$	M	VH	L	M	H	VL	L	H	VL
${C}_{22}$	M	VH	L	L	H	VL	M	H	VL
${C}_{31}$	M	H	VL	L	VH	VL	M	H	L
${C}_{32}$	M	M	L	VH	L	VL	H	H	VL
${D}_{11}$	M	VH	L	VH	H	VL	H	VL	L
${D}_{12}$	M	VH	H	VL	H	VL	VH	L	L
${D}_{21}$	M	VH	M	VL	H	L	VH	L	VL
${D}_{22}$	VH	VH	VL	L	H	L	M	H	VL
${D}_{31}$	VL	VL	M	VH	L	H	M	L	H
${D}_{32}$	L	VL	M	H	VL	VH	M	L	H

## References

[1] Foroughi S, Rasol MA (2016). Housing renovation priority in the fabric texture of the city using the analytic hierarchy model (AHP) and geographic information system (GIS): A case study of Zanjan City, Iran. Egypt. J. Remote Sens. Space Sci..

[2] Haghighi Fard SM, Doratli N (2022). Evaluation of resilience in historic urban areas by combining multi-criteria decision-making system and GIS, with sustainability and regeneration approach: The case study of Tehran (IRAN). Sustainability.

[3] Martí P, García-Mayor C, Serrano-Estrada L (2019). Identifying opportunity places for urban regeneration through LBSNs. Cities.

[4] Li YQ, Pan YH (2021). Research on renovation potential calculation and renewal scheme determination of old residential quarters. Constr. Econ..

[5] Ji J, Gao XL (2011). Method for evaluation environmental efficiency in urban residential areas. China Land Sci..

[6] Wu WH, Shi HJ, Yang BH, Xu YT, Li Y (2021). An analysis framework on enterprise communities' renewal potential of land use in the city and its application. Acta Geogr. Sin..

[7] Tao HY, Zhou SL, Zhou L (2014). Group decision-making on well-order renovation of urban villages: A case study of Guangzhou. Geogr. Res..

[8] Andersen R, Jensen LB, Ryberg M (2021). Using digitized public accessible building data to assess the renovation potential of existing building stock in a sustainable urban perspective. Sustain. Cities Soc..

[9] Ruá MJ, Huedo P, Cabeza M, Saez B, Agost-Felip R (2021). A model to prioritise sustainable urban regeneration in vulnerable areas using SWOT and CAME methodologies. J. Hous. Built Environ..

[10] Knippschild R, Zöllter C (2021). Urban regeneration between cultural heritage preservation and revitalization: Experiences with a decision support tool in Eastern Germany. Land.

[11] He QQ, Li XS, Wan YZ (2021). Analysis of the priority of the reconstruction of old community based on SEM: Taking Shangyixincun community in Nanjing as an example. Constr. Econ..

[12] Lv F, Ding MY, Sun PJ (2019). Resident satisfaction-based updating strategies of old communities: A case study of harbin demonstration communities. Areal Res. Dev..

[13] Serrano-Jiménez A, Lima ML, Molina-Huelva M, Barrios-Padura Á (2019). Promoting urban regeneration and aging in place: APRAM – An interdisciplinary method to support decision-making in building renovation. Sustain. Cities Soc..

[14] Bottero M, Oppio A, Bonardo M, Quaglia G (2019). Hybrid evaluation approaches for urban regeneration processes of landfills and industrial sites: The case of the Kwun Tong area in Hong Kong. Land Use Policy.

[15] Bottero M, D’Alpaos C, Oppio A (2018). Multicriteria evaluation of urban regeneration processes: An application of PROMETHEE method in Northern Italy. Adv. Oper. Res..

[16] Lee GKL, Chan EHW (2008). The analytic hierarchy process (AHP) approach for assessment of urban renewal proposals. Soc. Indic. Res..

[17] Zhang XD, Hu JC, Yang Q, Lin YL (2017). Research on the evaluation system of renewal for old residential district based on AHM and comprehensive assessment method. Urban Dev. Stud..

[18] Xiao Y, Chen J, Liu B (2019). Research on the implementation effect evaluation of old community upgrading and renovation. Constr. Econ..

[19] Zhu SY, Li DZ, Feng HB, Gu TT, Zhu JW (2019). AHP-TOPSIS-based evaluation of the relative performance of multiple neighborhood renewal projects: A case study in Nanjing, China. Sustainability.

[20] Li DZ, Zhu JW, Zhu SY (2020). Research on the lean governance evaluation of urban old neighborhood based on PCA-DEA Model. Mod. Urban Res..

[21] Lee JH, Lim S (2018). An analytic hierarchy process (AHP) approach for sustainable assessment of economy-based and community-based urban regeneration: The case of South Korea. Sustainability.

[22] Huo LY, Sun QQ, Hu HB (2019). The theory and practice of china's preschool education indicator system. Educ. Res..

[23] Li BM, Yan HB (2017). Teacher online training quality assessment based on CIPP evaluation model: A case study. Res. Educ. Dev..

[24] Kellaghan T, Stufflebeam DL (2003). International Handbook of Educational Evaluation.

[25] Luo H (2012). The research on equity index system of higher education based on CIPP model. Res. Educ. Dev..

[26] Wati S, Wijaya AF, Suryadi (2016). Evaluation of green open space management program in Gresik regency based on CIPP evaluation model. Jurnal Ilmiah Ilmu Administrasi Publik.

[27] Tao Y, Zhao SF, Hu X (2018). Entrepreneurial exit: A systematic literature review and directions for future research. Bus. Manag. J..

[28] Yang J, Wang GY, Liu Q, Guo YK, Liu Y, Gan WY, Liu YC (2018). Retrospect and prospect of research of normal cloud model. Chin. J. Comput..

[29] Li DY, Meng HJ, Shi XM (1995). Membership clouds and membership cloud generators. J. Comput. Res. Dev..

[30] Hu DB, Xie L (2021). Ecological environmental security evaluation based on cloud model and evidential reasoning. Oper. Res. Manag. Sci..

[31] Liu YF, Yu YX (2020). Analysis and improvement of PFMEA risk assessment based on optimal combination weights and cloud-VIKOR method. Appl. Res. Comput..

[32] Opricovic S, Tzeng GH (2007). Extended VIKOR method in comparison with outranking methods—ScienceDirect. Eur. J. Oper. Res..

[33] Devi K (2011). Extension of VIKOR method in intuitionistic fuzzy environment for robot selection. Expert Syst. Appl..

[34] Gao ZF, Yang Q, Peng DH (2013). Interval vikor multi-criteria decision method research based on cloud model. J. Kunming Univ. Sci. Technol. (Nat. Sci. Ed.).

[35] Li CB, Zhang L, Ma Y (2018). Multi-attribute group decision making with interval value based on cloud model and extension of VIKOR method for power grid enterprise's scientific and technological development. Oper. Res. Manag. Sci..

[36] Mo GL, Zhang WG, Liu F, Yu X (2019). Uncertain-language multi-criteria group decision making based on prospect-cloud model for stock indices investment in international wide. Oper. Res. Manag. Sci..

[37] Xu XH, Wu HD (2018). Approach for multi-attribute large group decision-making with linguistic preference information based on improved cloud model. J. Ind. Eng. Eng. Manag..

[38] Yang X, Yan L, Zeng L (2013). How to handle uncertainties in AHP: The Cloud Delphi hierarchical analysis. Inf. Sci..

[39] Wang JQ, Liu T (2012). Uncertain linguistic multi-criteria group decision-making approach based on integrated cloud. Control Decis..

[40] Jing SW, Tang Y, Yan JA (2018). The application of Fuzzy VIKOR for the design scheme selection in lean management. Math. Probl. Eng..

[41] Chan E, Lee GKL (2007). Critical factors for improving social sustainability of urban renewal projects. Soc. Indic. Res..

[42] Awad J, Jung C (2022). Extracting the planning elements for sustainable urban regeneration in Dubai with AHP (analytic hierarchy process). Sustain. Cities Soc..

[43] Wang CH, Li ZW (2019). Practice and exploration on the regeneration of old residential areas under the background of urban renewal: A case study of the regeneration of Zhonghua north village of Kunshan. Mod. Urban Res..

[44] Yung EHK, Conejos S, Chan EHW (2016). Social needs of the elderly and active aging in public open spaces in urban renewal. Cities.

